# Internal GFRP Reinforcement of Low-Grade Maritime Pine Duo Timber Beams

**DOI:** 10.3390/ma13030571

**Published:** 2020-01-24

**Authors:** Jose-Antonio Balmori, Luis-Alfonso Basterra, Luis Acuña

**Affiliations:** Research group of Timber Structures and Wood Technology, University of Valladolid, Avenida de Salamanca, 18, 47014 Valladolid, Spain; basterra@arq.uva.es (L.-A.B.); maderas@iaf.uva.es (L.A.)

**Keywords:** strengthening, low-grade timber, glass fiber-reinforced polymer (GFRP), duo timber beam, *Pinus pinaster* Ait. wood

## Abstract

This paper presented an experimental structural-scale test campaign used to analyze the flexural behavior of low-grade maritime pine (*Pinus pinaster* Ait.) duo timber beams reinforced with an internal glass fiber-reinforced polymer (GFRP) sheet. For this purpose, thirty (30) unreinforced duo beams and thirty (30) duo beams internally reinforced with a unidirectional GFRP sheet with an areal mass of 1200 g/m^2^ were produced and tested. The addition of a low GFRP reinforcement ratio (1.07%) in the tension zone of the duo beams provided an average improvement of 8.37% in bending stiffness (EI) and an increase of up to 18.45% in ultimate moment capacity. In addition to this improved bending behavior, the internal GFRP reinforcements seemed to decrease the influence of wood singularities and wood heterogeneity on mechanical properties, which allowed for better characteristic values to be reached and for the achievement of results with less variability.

## 1. Introduction

Timber has been traditionally used in construction for its excellent mechanical properties, high availability, lightness, and ease of work. In recent times, timber has also been valued as a renewable natural resource and an environmentally sustainable construction material [1]. For these reasons, the use of structural wood in buildings has increased exponentially in recent decades, especially industrialized wood products, such as timber glulam or cross-laminated timber (CLT) [2]. In this way, timber has become a highly attractive material for architects and engineers, increasing its demand and forcing sawmill companies to manage the entire production chain and optimize their production process [3]. This growing demand of timber has encouraged researchers to produce new wood-based products using low-grade timber from fast-growing species that are not currently implemented for structural use [4] and to optimize the use of lower quality woods that are rejected under current standards [5] to guarantee a sufficient production quality to meet the present and future timber demand.

The use of fiber-reinforced polymer (FRP) reinforcements to strengthen or repair timber products increases the stiffness and resistance of structural sections and reduces the variation in the mechanical properties [6]. FRPs have been extensively used in the renovation and repair of civil structures comprising a widely kind of materials, and research on timber beams started in the 1960s [7,8]. In recent times, FRP reinforcements have been deeply applied to strengthen and retrofit timber structures [9,10,11,12,13,14]. However, the development of new hybrid FRP-timber products is very scarce and limited [15,16,17,18]. FRP reinforcements have some advantages over other traditional reinforcement materials, such as steel, in terms of properties and durability. FRP has high mechanical properties (strength and stiffness) in relation to its weight, which is approximately four times less than steel. Additionally, FRPs are resistant to corrosion, and some are poor electrical conductors [19]. Among all FRP products, unidirectional glass fiber-reinforced polymers (GFRPs), especially E-Glass fibers, seems to be the most effective reinforcement for timber beams because of their low cost and suitable mechanical properties [20].

In the present study, duo timber beams were analyzed because they represent the most basic industrialization system [21]. New reinforced duo timber beams of maritime pine wood were produced and bending tested. During duo beam manufacturing, a rigid GFRP sheet was introduced inside the glue line in a vertical position. It was known that this orientation is less structurally efficient than other solutions; however, it had some advantages. This arrangement allowed us to make duo timber beams with the same global section but with different levels of performance by changing the internal reinforcement ratio without any visual impact. In addition, the position of the reinforcement protected by timber improved the fire resistance of the reinforcement [22] compared to other external FRP reinforcement configurations [23]. In this way, several researchers have advised to protect FRP reinforcements against exposure to high temperatures due to their limited stability in such conditions [24]. Despite these advantages, there have been few studies that used internal and vertical GFRP reinforcements, as placed and tested in this research. This may be because other configurations closer to the tension face are much more structurally efficient.

### 1.1. State of the Art

The first studies on the reinforcement of bending timber beams began with externally bonded FRP materials. Theakston [25] was one of the first researchers to demonstrate that GFRP reinforcement provided improved stiffness, strength, and ductility. This and other initial studies performed on small and clean wood pieces achieved improvements over 40% in ultimate strength and 20% in stiffness [26,27]. Since the 1990s, FRP-reinforced timber studies have increased, in which structural sized beams of different qualities were also tested. Triantafillou [28] made a large test campaign over sawn timber beams reinforced with external FRP materials and observed a significant increase in strength, stiffness, and ductility. Dagher et al. [29] tested glued laminated beams of different wood grades with varying types and GFRP ratios from 0.3% to 3.1%. Their results showed improvements of approximately 24%–51% in strength and approximately 25%–37% in stiffness, and they concluded that low-grade wood reinforced with GFRP could be competitive with higher-quality timber. The same conclusion was drawn by Tingley [30], who, before testing FRP-reinforced timber beams, also proposed the use of low-grade wood with FRPs as an alternative to high-grade wood. Following this line of thought, many researchers have compared external CFRP and GFRP reinforcements in structural sized sawn wood beams [31,32,33]. These studies achieved strength increases from 40% to 100% depending on the type and amount of reinforcement used. In addition, Parvez [34] compared different FRP reinforcements applied to sawn wood beams and concluded that GFRP reinforcements provide better cost-performance ratios in terms of flexural strength than CFRP reinforcements.

Despite these promising results, external FRP reinforcements have significant disadvantages regarding their structural application, such as visual impact, mechanical damage, and, more importantly, fire sensitivity [35]. For that reason, another line of research has been conducted on the internal FRP reinforcement of wood using bars and sheets with different configurations [36,37]. Following this approach, Raftery and Harte [38] proposed the introduction of GFRP in low-grade glulam timber beams with low reinforcement ratios between 1.10% and 1.25%. In addition, Fiorelli and Alves [39] presented a test campaign for laminated beams reinforced with low ratios of GFRP (1.2%–3.3%) in the last glue line. Their results showed a bending strength improvement close to 30%. Similar results were achieved by Raftery and Whelan [40], who studied the internal reinforcement of low-grade wood laminated beams with GFRP bars with a reinforcement ratio of 1.4% in the tension zone, which increased the flexural stiffness between 11% and 14%. Furthermore, the flexural stiffness increased up to 22%–29% by placing additional reinforcement in the compression zone. In addition to this bending strength and stiffness improvement, the results published by Gentile [41] showed that GFRP reinforcement allowed us to homogenize the beam section, compensating for local defects in the wood. Nevertheless, few studies have tested low-grade timber from the maritime pine (*Pinus pinaster* Ait.) wood species, and GFRP has never been tested vertically inside the beam section. Thus, Nadir [42] tested low-grade maritime pine timber reinforced with external GFRP and CFRP sheets of different thicknesses in the tension zone. Ribeiro [43] proposed an internal GFRP-reinforced laminated beam of maritime pine, which provided a promising 20% increase in ultimate strength and a 40% increase in bending stiffness.

### 1.2. Objectives

The motivation of this paper started with a research project whose main objective was to develop structural use for low-grade, low-cost, rejected timber from sustainable fast-growing forests, such as reforestation plantations of maritime pine (*Pinus pinaster* Ait.). This wood species, which has been widely used in poor ground reforestation, grows irregular trunks and generates a large volume of low-quality, rejected wood with low and uncompetitive stiffness and strength in sawmills. For this reason, this wood species is barely used for structural purposes, and it is currently allocated to secondary uses such as boards. This change of use may generate new economic resources, creating employment, and the establishment of population in rural areas. To reach this general objective, it is proposed to develop a new industrial product based on timber, improving its performance with GFRP reinforcements, which offer a sufficient relation between mechanical properties and cost.

The specific objective of this study is to examine the improvement efficiencies in the mechanical properties of low-grade timber, with a visual grading of non-structural wood quality, of maritime pine using an internal reinforcement for duo beams with low ratios of rigid GFRP sheets placed vertically within the section. For this purpose, we compare the performance of unreinforced and reinforced duo timber beams in terms of the load-deflection behavior, bending stiffness, and ultimate moment capacity.

## 2. Materials and Methods

### 2.1. Timber

This research used low-grade conifer timber of visual grading of non-structural wood quality according to standard EN 56544:2011 [44] from maritime pine, which was purchased from the same sawmill and produced from the batch in a Sierra de Oña forest (Burgos, Spain). Timber planks with a length of 2500 mm and a nominal section of 60 × 140 mm were sawn. This timber batch was maintained in the sawmill under natural hygrothermal conditions until the wood moisture content, measured by electrical resistance according to standard EN 13183-2 [45], was less than 18%. Once the timber planks arrived at the laboratory, they were conditioned inside climatic chambers until the humidity dropped to 12%.

The main timber properties and the corresponding standards used to obtain these properties are summarized in Table 1. The modulus of elasticity (MOE) was calculated without considering the shear deformation. 

The values shown in Table 1 are closer to those in other previous works on the mechanical properties of maritime pine [50,51].

### 2.2. GFRP Reinforcements

**Figure 1:**
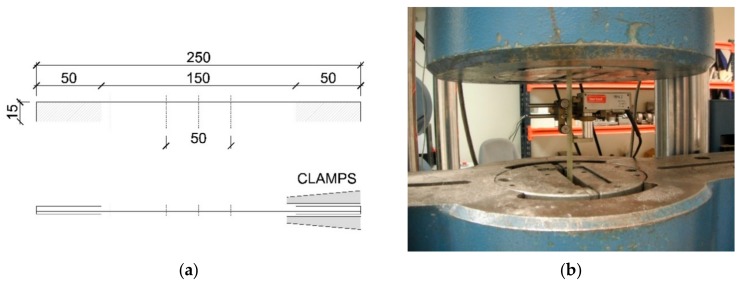
Glass fiber-reinforced polymer (GFRP) tensile specimens according to ISO 527/5: (**a**) specimen dimensions and (**b**) tensile test.

Twenty (20) specimens of rigid GFRP sheets with a nominal section of 2.15 × 15 mm and 250 mm of total length were produced using infusion techniques. For that, unidirectional 'E-Glass' fibers were embedded using high-pressure vacuum in an isophthalic unsaturated polyester resin. At the heads of test specimens, 50 mm beads were placed to avoid deterioration when the jaws of the test machine were tightened (Figure 1a). The UNI-1200 GFRP sheets (UNI-1200 indicates unidirectional fibers with an areal mass of 1200 g/m^2^) manufactured in this way, which have an areal mass of 1200 g/m^2^ and a mean thickness of 2.15 mm, were subjected to tensile tests in accordance with ISO 527-5/B [52] using an INSTRON MEN-102/100 testing machine equipped with a 1000 kN load cell and pneumatic clamps. Strain was measured with an IBERTEST IB-MFA 2 strain gauge with a 2 mm span (Figure 1b). A displacement rate of 1 mm/min was employed during testing.

The results reached [53] were a mean Young’s modulus of 21,610 MPa (CoV 4.43%) and a mean ultimate tensile strength of 455 Mpa (CoV 6.26%) for the UNI-1200 GFRP sheet.

### 2.3. Adhesive

A solvent-free and thixotropic two component structural adhesive, based on a mixture of epoxy resin and special filler (Sikadur-30), was used to manufacture the glued duo timber beams. In Table 2, the main mechanical properties supplied by adhesive manufacture are summarized. Although this adhesive has been widely used in civil engineering, an extensive process of shear tests and pull-off tests were carried out in accordance with standard ISO 6238 [54] and ISO 4624 [55] to verify the proper behavior of the bond between the wood planks and GFRP reinforcement sheets.

### 2.4. Specimen Preparation

**Figure 2:**
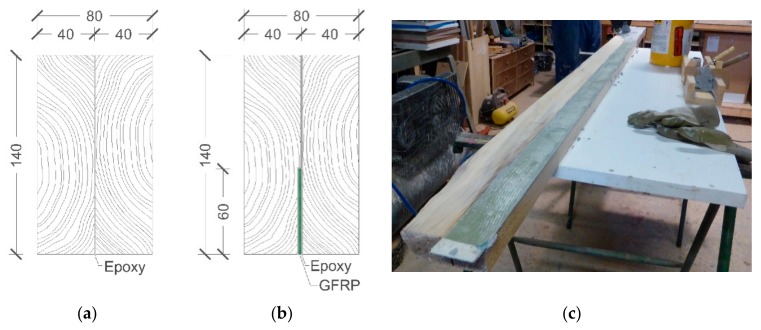
Duo beam section configurations with dimensions in mm: (**a**) unreinforced duo beam and (**b**) duo beam reinforced with a UNI-1200 GFRP sheet; (**c**) recess made on a board to lodge the GFRP.

For each duo beam type, thirty (30) specimens were manufactured in a carpentry workshop. The planks from humidity-conditioned stock were first cut with nominal dimensions of 40 × 140 × 2500 mm (Figure 2a,b) and later prepared via knife plane work at least 48 hours before bonding. In addition, a recess was cut on one of the timber planks to lodge the GFRP sheet (Figure 2c) without changing the external dimensions of the reinforced duo beam. Later, both planks were bonded with epoxy resin Sikadur-30 to produce duo timber beams, including a UNI-1200 GFRP sheet inside the glue line in a vertical position, only in the reinforced duo beams. The epoxy resin temperature was monitored throughout the gluing process to control the maximum pot life of the mixed resin. Over assembled duo timber beams were applied a constant pressure of 0.5 Mpa during a curing time of 48 h. The mean amount of epoxy resin applied was 1500 g/m^2^.

The area of the UNI-1200 GFRP reinforcement was 1.07% of the total duo beam section. An elastic modulus ratio between reinforcement and timber (E_f_/MOE) of 2.02 was calculated using the transformed section method. The GFRP sheet used developed a theoretical increase of 1.37% in the bending stiffness (EI) and a theoretical increase of 2.14% in the ultimate moment capacity (M_ult_) of the reinforced duo timber beams.

### 2.5. Experimental Setup

**Figure 3:**
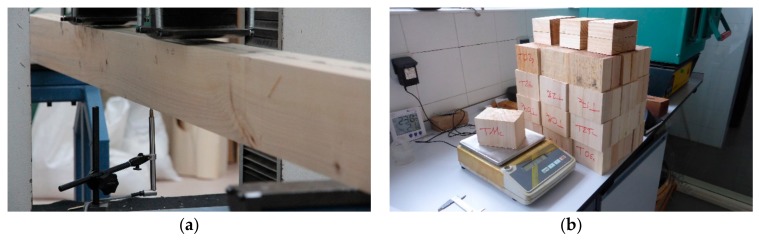
Experimental setup established according to standard EN 408: (**a**) linear variable differential transformer (LVDT) recording deformation during the bending tests and (**b**) humidity-controlled specimen extracted from each tested duo beam in accordance with standard EN 13183-1.

Both unreinforced and reinforced duo beams were tested according to standard EN 408 [47] under a four-point bending test. The distance between supports was 2200 mm, whereas the distance between central loading points was 840 mm. A constant displacement rate of 12 mm/min was employed during testing. Deformations were measured at the center of the beams with a linear variable differential transformer (LVDT) (Figure 3a). The global bending modulus of elasticity was calculated from a stress–strain curve in a range between 10% and 40% of the estimated ultimate load. During tests, LVDT was retired when the load reached 40% of the estimated ultimate load, and later the test proceeded until beam failure. All beams were broken after 300 ± 120 s. After each beam test, the failure mode was examined, and its type, position, and features were annotated.

After finishing the test campaign, a specimen with a length of 80 mm was sawn from each broken beam (Figure 3b), and the real moisture content and wood density were obtained by the oven drying method in accordance with EN 13183-1 [56]. With this real moisture content values, the bending modulus of elasticity of each beam was corrected according to factors proposed in standard EN 384 [46]. 

## 3. Results and Discussion

### 3.1. Failure Modes and Load-deflection Behavior

All duo beams tested, both reinforced and unreinforced, exhibited mainly linear elastic behavior until failure, except for in their initial and their final ranges of loading. Both types of duo beams exhibited tensile failure, and in all of them, the failure started in defects or irregularities of the wood (knots, fiber deviation, etc.). After each test, a visual breakage analysis was performed. No plasticization in the compression zone and no bond failures were observed.

**Figure 4:**
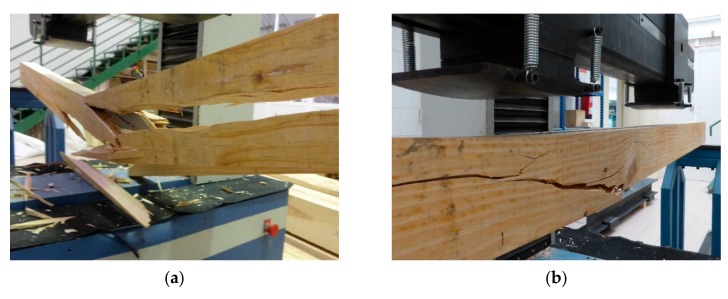

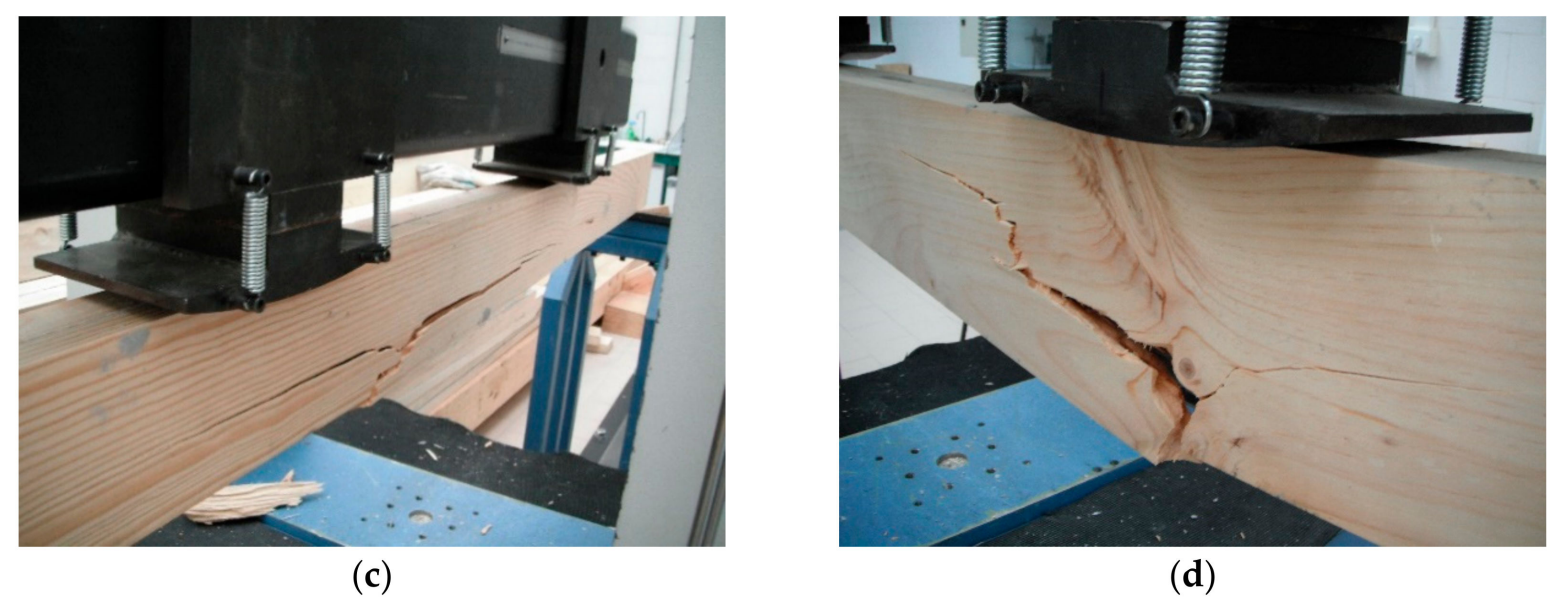
Typical fracture of maritime pine duo beams under bending tests: (**a** and **b**) unreinforced duo beam at failure and (**c** and **d**) duo beam reinforced with a UNI-1200 GFRP sheet at failure.

The maritime pine had widespread knots, with large knots and skipping knots. Due to the recurring presence of knots, several failures were associated with fiber deflection in traction zones. This typical breakage pattern of unreinforced beams is shown in Figure 4a,b. According to the wood properties (Table 1), the theoretical ultimate deflection was calculated by assuming linear elastic behavior and the theoretical values of the bending stiffness (EI). The fracture of the 80 × 140 mm unreinforced duo beams occurred at a load of 28.83 kN with a theoretical deformation of 41.59 mm and a mean experimental deformation of 44.55 mm (increase of 7.12%) (Table 3). 

The comparison of the ultimate load and deflection of duo beams in Table 3 shows that the UNI-1200 GFRP reinforcement placed in the tension zone of the maritime pine duo beams significantly increased the ultimate load capacity (15.09%), providing a mean ultimate load of 33.18 kN, a theoretical deformation of 48.48 mm, and a mean experimental deformation of 55.43 mm (increase of 14.34%). In addition, the ultimate deformation increased moderately from 44.55 mm for the unreinforced duo beams to 55.43 mm for the reinforced beams (24.42%) because it was limited by the tensile failure of the wood. Reinforced beams had larger ultimate deformation than unreinforced beams because the GFRP sheet placed inside the glue-line limited the influence of the defects or irregularities in the wood, and this allowed greater tensile strains and stresses in the beam to be reached.

The failure analysis of the reinforced beams was similar to that of the unreinforced duo beams, in which the specimens primarily exhibited tensile failure (Figure 4c,d). Plasticization damage was not observed in the compression zone of the reinforced duo beams, and the GFRP-wood bond showed proper behavior without any adhesion failures. This improvement in the reinforced timber beams with GFRP sheets is similar to that from other results in the literature [38,39,40,41].

### 3.2. Bending Stiffness

**Figure 5:**
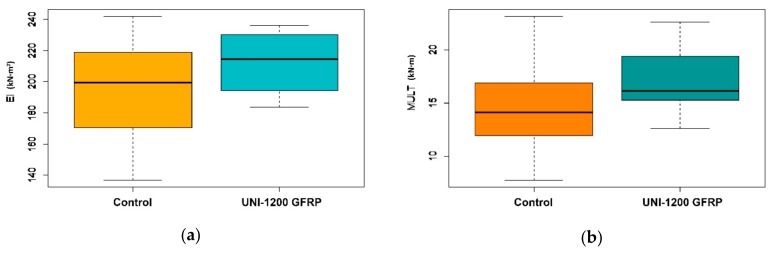
Box plot graphics of mechanical properties: (**a**) bending stiffness EI (kN·m^2^) and (**b**) ultimate moment capacity M_ult_ (kN·m). Note: A color-friendly palette (orange/blue) was used to assist color-blind readers.

Experimental bending stiffness (EI) values were obtained in the elastic deformation range from the load and the deformation measures of the beam at 10% and at 40% of its ultimate capacity. Theoretical stiffness values were obtained by the transformed section method [34,57]. Comparisons between the theoretical and experimental values are summarized in Table 4 and Figure 5a.

The reinforced duo beams exhibited significantly higher bending stiffness (8.37%) than the unreinforced beams. Moreover, the reinforced duo beams also exhibited a lower dispersion in their results (CoV 8.16%) than the unreinforced duo beams (CoV 14.97%). This lower variation meant that the characteristic values (5^th^ percentile) of the reinforced duo beams increased even with similar mean values. These results were comparable to those reported in other studies involving GRFP with similar reinforcement ratios. Thus, Raftery [38], who used GFRP reinforcement ratios of 1.7%–3%, obtained stiffness increases of 10%–12%, which is very similar to the improvement ranges shown in Table 4. Fiorelli [39], who used GFRP reinforcement ratios of 1%–3%, obtained a stiffness increase of 13%–42%. In tests with maritime pine timber beams, Ribeiro [43] obtained a stiffness increase of 20%–43% with GFRP reinforcement ratios of 1.2%–4.2%. However, note that all these referenced studies, despite using GFRP laminates, used varied reinforcement configurations in their tests.

### 3.3. Ultimate Moment Capacity

The ultimate moments (M_ult_) were calculated for each test specimen with Equation (1), where *F_ult_* is the ultimate breaking force (kN) reached in the test and *d* is the distance between the support and the loading point.

M_ult_ = 0.5·F_ult_·d (1)

**Figure 6:**
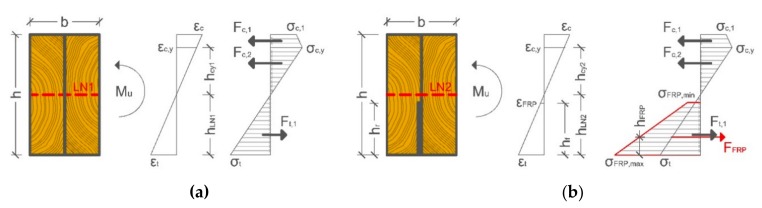
Bazan–Buchanan model of bending stress distribution: (**a**) unreinforced duo beam and (**b**) duo beam reinforced with a UNI-1200 GFRP sheet. *LN* is location of the neutral axis for unreinforced (1) and reinforced (2); *ε_c_*, *ε_cy_*, and *ε_t_* are wood compression and tensile strains; *ε_FRP_* is reinforcement tensile strain; *F_c,1_, F_c,2_*, and *F_t,1_* are compressive and tensile forces in wood; F_FRP_ is tensile force in the reinforcement; *h* is different height references; *σ_c,1_,* *σ_c,y_*, and *σ_t_* are compression and tensile stresses in wood; *σ_FRP, min_* and *σ_FRP, max_* are stresses in reinforcement.

The theoretical values of the ultimate moment were obtained by the Bazan–Buchanan model [8,58,59], as seen in Figure 6, which assumes a nonlinear stress distribution in compression. A comparison between the theoretical values and experimental results is presented in Table 5.

Figure 5b shows the ultimate moment capacity results with maximum, minimum, average, and quartile (25% and 75%) data ranges. The data show a great improvement in M_ult_ (18.45%) and a lower dispersion (CoV 15.96%) than the unreinforced duo beams (CoV 25.88%). Moreover, the experimental results were higher than the theoretical values because the GFRP sheets seemed to reduce the influence of timber heterogeneity and timber singularities.

The results of the reinforced beams presented a significantly improved ultimate moment (18.45%) and a lower dispersion. This improvement was similar to that reported by Ribeiro [43] (25%–32%), who used similar GFRP reinforcement ratios. With higher reinforcement ratios (3%–6%), Corradi [60] obtained greater ultimate moment increases of 33%–79%.

### 3.4. Statistical Analysis

In the statistical analysis, the normality of the data was verified for all populations using the Shapiro–Wilks normality test and a Q–Q normal probability plot; hence, we were able to assume normality in all cases. The homoscedasticity requirement was analyzed via Levene's test, broken down for the different groups. Therefore, the Welch test was used to compare these groups. Based on the t-test and Welch test performed on the ultimate moment capacity and the bending stiffness, which were used to detect differences between the groups of beams, Table 6 confirms that there were significant differences (at 95%) between the control and reinforced groups.

The comparative analysis between the unreinforced and reinforced duo beams showed that there were statistically significant differences in both the bending stiffness and the ultimate moment capacity.

## 4. Conclusions

The results of a test campaign to reinforce low-grade maritime pine (*Pinus pinaster* Ait.) duo timber beams in bending using internal GFRP sheets were discussed. The final conclusions reached were as follows:

- Maritime pine duo beams with a low ratio (less than 2%) of GFRP reinforcements (unidirectional sheets with an areal mass of 1200 g/m^2^) attached on the tension zone exhibited higher bending stiffness and ultimate moment capacity than unreinforced duo beams.
- Using a modest reinforcement ratio of 1.07%, the GFRP sheets increased the bending stiffness by 8.37% and the ultimate moment by 18.45%.
- Theoretical increase in bending stiffness calculated by the transformed section method was over 1.37%, whereas the experimental improvement exceeded 8.37%. In terms of ultimate moment capacity, the theoretical increase was greater than 2.14%, whereas the experimental improvement exceeded 18.45%.
- Reinforced duo beams exhibited larger ultimate deformations, increasing from 44.55 mm in the unreinforced beams to 55.43 mm in the reinforced beams; as a result, higher tensile strains and stresses were achieved in the timber. The average ultimate stress in the timber increased by 24.42%.
- Reinforced duo beams could resist an ultimate load 1.15 times greater than unreinforced duo beams.
- The variation in mechanical properties decreased in the reinforced beams. This likely involved some reduction in the influence of defects and singularities in the timber.
- The low cost of GFRP sheets compared to other reinforcement types (for example CFRPs or BFRPs) and the achieved improvements in low-grade maritime pine duo beams from low reinforcement ratios (< 2%) show a possible commercial development of sustainable GFRP-reinforced timber duo beams.

## Figures and Tables

**Table 1:** Main properties of maritime pine tested.

Property	Mean Value	5th Perc. Value	Standard
Moisture content (%)	11.25 (7.63%)	9.58	EN 13183-2:2002 [45]
Density (kg/m^3^)	510 (16.85%)	480	EN 384:2016 [46]
Young’s modulus–MOE (MPa)	10,689 (14.70%)	7631	EN 408:2011 [47]
Rupture modulus–MOR (MPa)	54.75 (25.41%)	30.13	EN 408:2011 [47]
Tension parallel (MPa)	71.04 (11.34%)	63.32	ISO 13061-6:2014 [48]
Compression parallel (MPa)	42.50 (10.43%)	36.24	UNE 56535:1977 [49]

Notes: Values in parentheses are coefficients of variation (CoV).

**Table 2:** Main mechanical properties of epoxy adhesive Sikadur-30 (exposed by manufacturer).

Property	Mean Value	Standard
Shear strength (Mpa)	19	DIN 53483:1969
Compressive strength (Mpa)	80	ASTM C579:2018
Tensile strength (Mpa)	31	ISO 527:2012
Bending Modulus of elasticity (Mpa)	2000	ISO 178:2011

**Table 3:** Ultimate load and deflection of maritime pine beams.

Description	No.	Experimental Ultimate Load F_ult_ (kN) Mean ± SD	Theoretical Ultimate Deflection (mm)	Experimental Ultimate Deflection (mm) Mean ± SD
Unreinforced duo beam	30	28.83 ± 7.34	41.59	44.55 ± 11.48
Reinforced duo beam (UNI-1200 GFRP sheet)	30	33.18 ± 8.36	48.48	55.43 ± 12.49

**Table 4:** Mean value, improvement, and coefficient of variation for the bending stiffness (EI).

Description	No.	Theoretical EI (kN·m^2^)	Experimental EI (kN·m^2^) Mean ± SD	Improvement (%)	CoV (%)
Unreinforced duo beam	30	195.53	195.53 ± 29.26 ^a^	—	14.97
Reinforced duo beam (UNI-1200 GFRP sheet)	30	198.21	211.91 ± 17.30 ^b^	8.37	8.16

Notes: The different letters (a, b) indicate significant differences in the Tukey post hoc test (p< 0.05).

**Table 5:** Mean value, improvement, and coefficient of variation for the ultimate moment capacity.

Description	No.	Theoretical M_ult_ (kN·m)	Experimental M_ult_ (kN·m) Mean ± SD	Improvement (%)	CoV (%)
Unreinforced duo beam	30	10.73	14.31 ± 3.70 ^a^	—	25.88
Reinforced duo beam (UNI-1200 GFRP sheet)	30	10.96	16.95 ± 2.66 ^b^	18.45	15.96

Notes: The different letters (a, b) indicate significant differences in the Tukey post hoc test (p< 0.05).

**Table 6:** Welch test performed on bending stiffnesses and ultimate moment capacities.

Group		No.	Mean Value	Levene Test Homoscedasticity	p-value Comparison Groups
EI (kN·m^2^)	Control	30	195.53 ^a^	0.0159	0.0157 (Welch)
	Reinforced	30	211.91 ^b^		
M_ult_ (kN·m)	Control	30	14.31 ^A^	0.219	0.005 (t test)
	Reinforced	30	16.95 ^B^		

Notes: Different letters (a, b) and (A, B) indicate significant differences (p< 0.05).

## References

[1] Anderson J., Edwards S., Mundy J., Bonfield P. (2008). Life Cycle Impacts of Timber: A Review of the Environmental Impacts of Wood Products in Construction.

[2] Thelandersson S., Larsen H.J. (2003). Timber Engineering.

[3] Brazier J.D. (1977). The effect of forest practices on quality of harvested crop. For. Int. J. For. Res..

[4] Castro G., Paganini F. (2003). Mixed glued laminated timber of poplar and Eucalyptus grandis clones. Holz Roh. Werkst..

[5] Johansson G., Kliger R., Perstorper M. (1994). Quality of structural timber-product specification system required by end-users. Holz Roh. Werkst..

[6] Parvez A. (2004). The Reinforcement of Timber for Structural Applications and Repair. Ph.D. Thesis.

[7] Bakis C.E., Bank L.C., Brown V.L., Cosenza E., Davalos J.L., Lesko J.J., Machida A., Rizkalla S.H., Triantafillou T.C. (2002). Fibre-Reinforced polymer composites for construction—State of the art review. J. Compos. Constr..

[8] Schober K.U., Harte A.M., Kloger R., Jockwer R., Xu Q., Chen J.F. (2015). FRP reinforcement of timber structures. Constr. Build. Mater..

[9] Borri A., Corradi M., Grazini A. (2005). A method for flexural reinforcement of old wood beams with CFRP materials. Compos. Part B.

[10] De Jesús A., Pinto J., Morais J. (2012). Analysis of solid wood beams strengthened with CFRP laminates of distinct lengths. Constr. Build. Mater..

[11] Nowak T.P., Jasienko J., Czepizák D. (2013). Experimental tests and numerical analysis of historic bent timber elements reinforced with CFRP strips. Constr. Build. Mater..

[12] Corradi M., Borri A., Castori G., Speranzini E. (2016). Fully Reversible Reinforcement of Softwood Beams with Unbonded Composite Plates. Compos. Struct..

[13] De la Rosa P., Cobo A., González M. (2016). Analysis of the flexural stiffness of timber beams reinforced with carbon and basalt composite materials. Compos. Part B.

[14] Gómez E.P., González M.N., Hosokawa K., Cobo A. (2019). Experimental study of flexural behavior of timber beams reinforced with different kinds of FRP and metallic fibers. Compos. Struct..

[15] Raftery G.M., Rodd P.D. (2015). FRP reinforcement of low-grade glulam timber bonded with wood adhesive. Constr. Build. Mater..

[16] Yang H., Liu W., Lu W., Zhu S., Geng Q. (2016). Flexural behavior of FRP and steel reinforced glulam beams: Experimental and theoretical evaluation. Constr. Build. Mater..

[17] Thorhallsson E.R., Hinriksson G.I., Snæbjörnsson J.T. (2017). Strength and stiffness of glulam beams reinforced with glass and basalt fibres. Compos. Part B.

[18] O’Ceallaigh C., Sikora K., McPolin D., Harte A.M. (2019). The mechano-sorptive creep behavior of basalt FRP reinforced timber elements in a variable climate. Eng. Struct..

[19] Féderation International Du Béton (2001). Design and use of externally bonded FRP reinforcement for RC structures. Bulletin.

[20] Basterra L.A., Acuña L., Casado M., López G., Bueno A. (2012). Strength testing of Poplar duo beams, *Populus x euramericana* (Dode) Guinier cv. I-214, with fibre reinforcement. Constr. Build. Mater..

[21] Müller C. (2000). Holzleimbau. Laminated Timber Construction.

[22] Martin Z.A., Tingley D.A. Fire Resistance of FRP Reinforced Glulam Beams. Proceedings of the World Conference on Timber Engineering.

[23] Council N.R. (2007). Guidelines for the Design and Construction of Externally Bonded FRP Systems for Strengthening Existing Structures.

[24] Zigler R., Pokorny M. (2015). Fire protection of timber structures strengthened with FRP materials. Civ. Eng. J..

[25] Theakston F.H. (1965). A Feasibility Study for Strengthening Timber Beams with Fibreglass. Can. Agric. Eng..

[26] Bulleit W.M. (1983). Reinforcement of wood materials: A review. Wood Fiber Sci..

[27] Rowlands R.E., Van Deweghe R.P., Laufenberg T.L., Krueger G.P. (1986). Fibre-reinforced wood composites. Wood Fiber sci..

[28] Triantafillou T.C., Deskovic N. (1992). Prestressed FRP sheets as external reinforcement of wood members. J. Mater. Civ. Eng..

[29] Dagher H.J., Kimball T.E., Shaler S.M., Abdel-Magid B. Effect of FRP Reinforcement on Low Grade Eastern Hemlock Glulam. National. Proceedings of the National Conference on Wood Transportation Structures.

[30] Tingley D.A. (1996). The Stress-Strain Relationships in Wood and Fibre-Reinforced Plastic Laminae of Reinforced Glued-Laminated Wood Beams. Ph.D. Thesis.

[31] Johns K.C., Lacroix S. (2000). Composite reinforcement of timber in bending. Can. J. Civ. Eng..

[32] Fiorelli J., Alves Dias A. (2003). Analysis of the Strength and Stiffness of Timber Beams Reinforced with Carbon Fibre and Glass Fibre. Mater. Res..

[33] Rescalvo F.J., Valverde-Palacios I., Suarez E., Gallego A. (2018). Experimental and analytical analysis for bending load capacity of old timber beams with defects when reinforced with carbon fibre strips. Materials.

[34] Parvez A., Ansell M., Smedley D. (2009). Mechanical repair of timber beams fractured in flexure using bonded-in reinforcements. Compos. Part B.

[35] Avent R., Issa C.A. (1984). Effect of fire on epoxy-repaired timber. J. Struct. Eng..

[36] Kliger R., Haghani R., Brunner M., Harte A., Schober K.U. (2016). Wood-based beams strengthened with FRP laminates: Improved performance with pre-stressed systems. Eur. J. Wood Wood Prod..

[37] Rescalvo F.J., Valverde-Palacios I., Suarez E., Gallego A. (2017). Experimental Comparison of Different Carbon Fibre Composites in Reinforcement Layouts for Wooden Beams of Historical Buildings. Materials.

[38] Raftery G.M., Harte A.M. (2011). Low-grade glued laminated timber reinforced with FRP plate. Compos. Part B.

[39] Fiorelli J., Alves Dias A. (2011). Glulam beams reinforced with FRP externally bonded: Theoretical and experimental evaluation. Mater. Struct..

[40] Raftery G., Whelan C. (2014). Low-grade glued laminated timber beams reinforced using improved arrangements of bonded-in GFRP rods. Constr. Build. Mater..

[41] Gentile C., Svecova D., Rizkalla S.H. (2002). Timber beams strengthened with GFRP bars: Development and applications. J. Compos. Constr..

[42] Nadir Y., Nagarajan P., Ameen M., Arif M. (2016). Flexural Stiffness and Strength Enhancement of Horizontally Glued Laminated Wood Beams with GFRP and CFRP Composite Sheets. Constr. Build. Mater..

[43] Ribeiro A., De Jesús A., Lima A., Lousada J. (2009). Study of strengthening solutions for glued-laminated wood beams of pine wood maritime. Constr. Build. Mater..

[44] UNE-EN 56544:2011 (2011). Visual Grading for Structural Sawn Timber. Coniferous Timber.

[45] UNE-EN 13183-2:2002 (2002). Moisture Content of a Piece of Sawn Timber. Part 2: Estimation by Electrical Resistance Method.

[46] UNE-EN 384:2016 +A1:2019 (2019). Structural timber—Determination of Characteristic Values of Mechanical Properties and Density.

[47] UNE-EN 408:2011+A1:2012 (2012). Timber structures–Structural Timber and Glued Laminated Timber. Determination of Some Physical and Mechanical Properties.

[48] ISO 13061-6:2014 (2014). Physical and Mechanical Properties of Wood—Test Methods for Small Clear Wood Specimens—Part 6: Determination of Ultimate Tensile Stress Parallel to Grain.

[49] UNE 56535:1977 (1977). Características Físico-Mecánicas de la Madera. Determinación de la Resistencia a Compression Axial.

[50] García-Iruela A., Esteban L., De Palacios P., García-Fernández F., De Miguel A., Vázquez E., Simón C. (2016). Resinous Wood of Pinus pinaster Ait.; Physio-mechanical properties. BioResources.

[51] Esteves B., Nunes L., Pereira H. (2011). Properties of furfurylated wood (Pinus pinaster). Eur. J. Wood Prod..

[52] ISO 527-5:2009 (2009). Plastics. Determination of Tensile Properties. Part 5: Test Conditions for Unidirectional Fibre-Reinforced Plastic Composites.

[53] Basterra L.A., Balmori J.A., Morillas L., Acuña L., Casado M. (2017). Internal reinforcement of laminated duo beams of low-grade timber with GFRP sheets. Constr. Build. Mater..

[54] ISO 6238:2018 (2018). Adhesives. Wood to Wood Adhesive Bonds. Determination of Shear Strength by Compressive Loading.

[55] ISO 4624:2016 (2016). Paints and Varnishes. Pull-Off Test for Adhesion.

[56] UNE-EN 13183-1:2019 (2019). Moisture Content of a Piece of Sawn Timber. Part 1: Determination by Oven Dry Method.

[57] Franke A., Franke B., Harte A.M. (2015). Failure modes and reinforcement techniques for timber beams—State of the art. Mater. Struct..

[58] Bazan I. (1980). Ultimate Bending Strength of Timber Beams. Ph.D. Thesis.

[59] Buchanan A.H. (1990). Bending strength of lumber. J. Struct. Eng..

[60] Corradi M., Thuc P., Poologanathan K., Osofero A. (2018). Flexural behavior of hardwood and softwood beams with mechanically GFRP plates. Compos. Struct..

